# One Versus Two Veins in Free Anterolateral Thigh Flap Reconstruction: A Systematic Review and Meta-Analysis

**DOI:** 10.7759/cureus.32358

**Published:** 2022-12-09

**Authors:** Evan O Wright, Shafiq Rahman

**Affiliations:** 1 Plastic Surgery, Pinderfields Hospital, Mid Yorkshire Hospitals NHS Trust, Leeds, GBR

**Keywords:** plastic and reconstructive surgery, anterolateral thigh (alt) flap, free alt flap, venous anastomosis, meta-analysis

## Abstract

There is considerable debate in the literature as to whether one or two venous anastomoses are optimal in the anterolateral thigh (ALT) free-flap reconstruction. The literature is currently devoid of a systematic review and meta-analysis of studies evaluating these procedures. This review will therefore be the first to address this clinical question. In accordance with the Preferred Reporting Items for Systematic Reviews and Meta-Analyses (PRISMA) guidelines, two authors (EW and SR) independently searched the following electronic databases: MEDLINE, EMBASE, CINAHL, and the Cochrane Central Register of Controlled Trials (CENTRAL). Case-control, randomised control and observational studies were included. The authors did not include case reports, case series, letters or abstracts. All patients were included regardless of age, co-morbidity status, and the anatomical site of reconstruction. Venous congestion/thrombosis, flap take-back rate due to venous insufficiency, flap loss and operative time were the primary outcome measures. Secondary outcome measures included partial flap loss and haematoma formation. The Newcastle Ottawa Scale was used to assess the risk of bias in the included studies. Review Manager 5.4 data synthesis software was used for the analysis. The authors identified eight observational studies, with a total of 1741 patients reviewed, demonstrating a significantly lower flap take-back rate for a double venous anastomosis and a shorter operative time in the single venous anastomosis group. However, other reported measures, including venous congestion and flap loss, showed a non-significant difference (P>0.05). The limitations of the evidence included in this review were that all studies were observational in design. The flap take-back rate is significantly less when anastomosing two veins, and the authors recommend that utilising a second vein can circumvent the caveat of venous compromise.

## Introduction and background

In plastic surgery, the anterolateral thigh (ALT) flap is a recognised versatile perforator flap. Initially advocated by Song et al. [1], it has a wide variety of uses, including reconstruction of the head and neck [2-4], breast [4], as well as limb defects [4]. There is considerable debate in the literature, however, associated with techniques of microvascular anastomosis [3] and whether one or two venous anastomoses are better. Authors advocating one vein for ALT flaps have emphasised lower operative times, improved resource optimisation and a reduced risk of thrombosis from a theoretically decreased blood flow velocity when two veins are anastomosed [5-7]. Utilising a second vein has been reported by others to mitigate the risk of potential flap compromise from venous failure [8,9]. To the authors’ knowledge, the literature is currently devoid of a systematic review and meta-analysis evaluating outcomes of studies comparing one versus two venous anastomoses in ALT-free flap reconstruction.

## Review

Methods

This meta-analysis and systematic review were performed as per the Preferred Reporting Items for Systematic Reviews and Meta-Analyses (PRISMA) statement standards (Appendices) [10]. The authors did not register the review protocol with the International Prospective Register of Systematic Reviews.

Eligibility Criteria

The aim was to include case-control, randomised control and observational studies that compared outcomes for one versus two venous anastomoses in ALT free-flap reconstruction. The authors did not include case reports, case series, letters or abstracts. Two venous anastomoses were the intervention of interest, with single venous anastomosis being the comparator as a control group. All patients were included regardless of age, co-morbidity status and the anatomical site of reconstruction. Studies not reported in English were excluded from the review.

Outcome Measures

Venous congestion/thrombosis, flap take-back rate due to venous insufficiency, flap loss and operative time were the primary outcome measures. Secondary outcome measures included partial flap loss as well as haematoma formation.

Literature Search

Two authors (EW and SR) independently searched the following electronic databases MEDLINE, EMBASE, CINAHL and the Cochrane Central Register of Controlled Trials (CENTRAL). The search strategy was developed by both authors and refined by the senior author (SR). Any discrepancies, at any stage, in the screening of the articles were resolved following discussion and re-evaluation by the authors. The last search was run on the 20th of September, 2022.

The search terminologies, as well as medical subject headings (MeSH), were combined with the adjuncts of "and" as well as "or" and included (“one vein”[All Fields] OR “single venous anastomosis”[All Fields]) AND (“two veins”[All Fields] OR “double venous anastomosis”[All Fields]) AND (“ALT flap”[All Fields} OR “anterolateral thigh flaps”[All Fields]) AND (“reconstruction”[All Fields]). Bibliographic lists of articles were also reviewed to enhance the screening process.

Study Selection

The titles and abstracts of articles that met the eligibility criteria within the literature were independently reviewed by two authors (EW and SR). Articles that met the eligibility criteria were then reviewed through their full text.

Data Collection

An electronic data extraction spreadsheet was created in accordance with Cochrane's data collection form concerning intervention reviews. Pilot testing was conducted in random articles and adjusted accordingly. Two authors (EW and SR) independently collated and entered the data into the spreadsheet.

Methodological Quality and Risk of Bias Assessment

Two authors (EW and SR) independently assessed the methodological quality and bias risk for included articles. The Newcastle Ottawa Scale was used to review the methodological quality and risk of bias for all non-randomised trials or observational studies [11]. It uses a star system with three domains: selection, comparability and exposure. Scores of nine are considered low risk of bias, those between seven and eight are regarded as medium risk and a score of six or lower is a high risk indicator of bias.

Data Synthesis and Statistical Analyses

The odds ratio (OR) was used for dichotomous variables representing the odds of an event in the double venous anastomosis group compared to the single vein group. Review Manager 5.4 data synthesis software (Review Manager (RevMan) (Computer program). Version 5.4, The Cochrane Collaboration, 2020) was used for the analysis. The senior author (SR) has experience in performing meta-analyses using Review Manager 5.4. Results were reported in a forest plot with 95% confidence intervals (CIs).

Heterogeneity among the studies was assessed using the Cochran Q test (χ2) as well as the I2 and interpreted as follows: 0% to 25% (low heterogeneity), 25% to 75% (moderate heterogeneity) and 75% to 100% (considerable heterogeneity).

Results 

Literature Search Results

**Figure 1:**
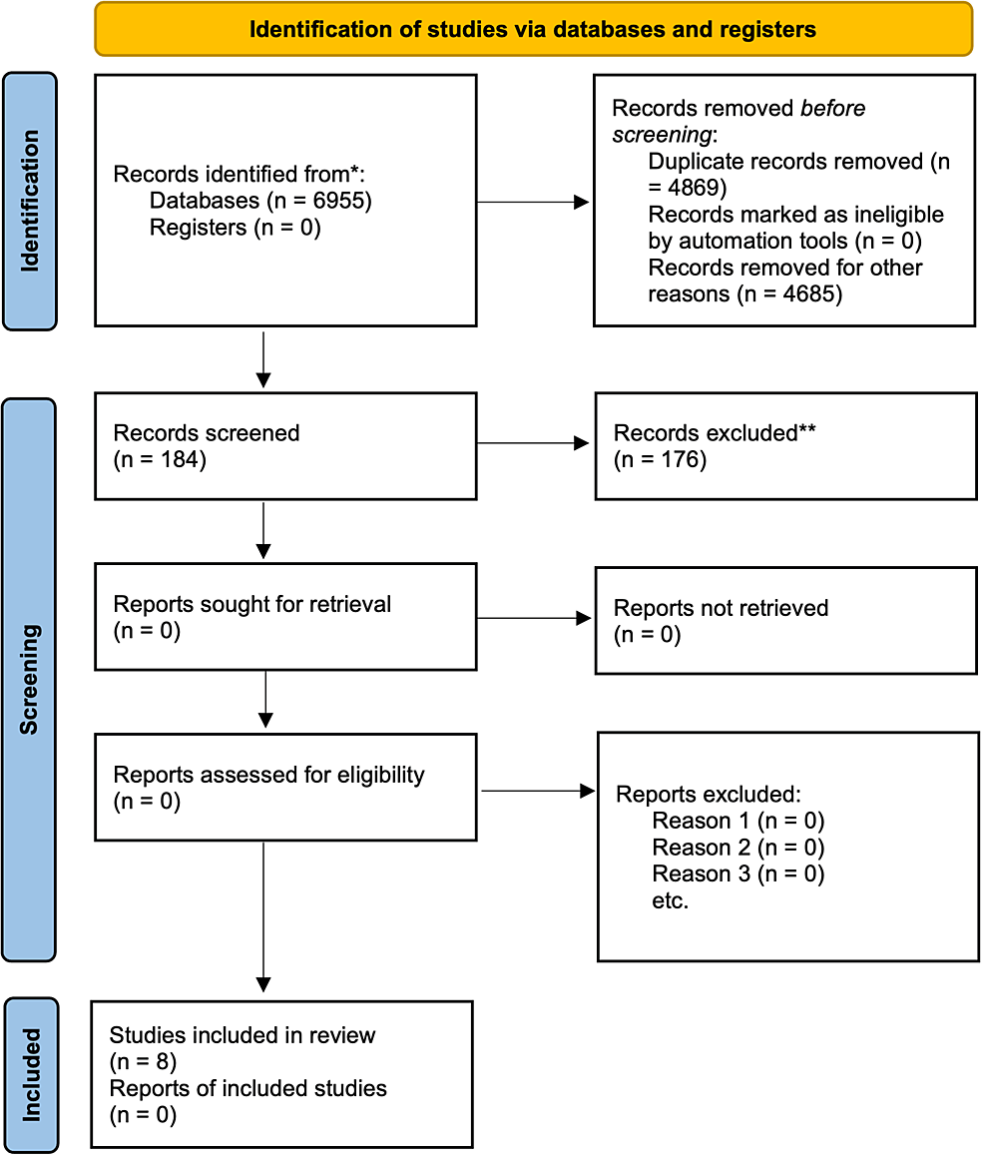
PRISMA flow diagram for article screening and selection comparing one versus two venous anastomoses in ALT free flap reconstruction PRISMA: Preferred Reporting Items for Systematic Reviews and Meta-Analyses; ALT: anterolateral thigh

Our database and registers search identified 6955 studies. Following a meticulous screening process and study exclusion, the authors identified eight studies for review (Figure 1, Table 1).

**Table 1:** Summary of selected studies including year, study design, sex, number of anastomoses and anatomical region reconstructed

Study	Year	Study design	M:F	One vein (n)	Two veins (n)	Anatomical region reconstructed
Ross et al [8]	2008	Observational	323:196	345	147	Head and neck
Lee et al [12]	2016	Observational	309:11	192	129	Intraoral
Ehrl et al [13]	2017	Observational	55:24	32	54	Upper limb
Iamaguchi et al [14]	2019	Observational	32:6	17	21	Limb
Abdelaal et al [15]	2019	Observational	45:15	35	25	Lower limb
Heidekrueger et al [16]	2016	Observational	239:115	141	213	Lower limb
Chen et al [17]	2013	Observational	303:12	195	120	Head and neck
Lin et al [18]	2009	Observational	55:1	80	32	Intraoral

Primary Outcome Measures

**Figure 2:**
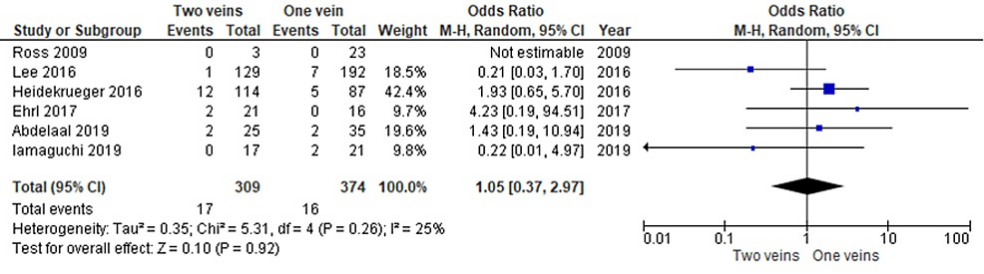
Total flap loss rate comparing one versus two venous anastomoses for ALT flaps. No significant difference identified between the two groups. Data reported by Ross, Lee, Heidekrueger, Ehrl, Abdelaal and Imaguchi et al. [8,12-16].

Flap loss rate: Flap loss rate was reported by six studies [8,12-16] in total, with comparable results between one versus two venous anastomoses in ALT flap reconstruction. No significant difference was seen with an odds ratio assessment (P > 0.5), as demonstrated in Figure 2.

**Figure 3:**
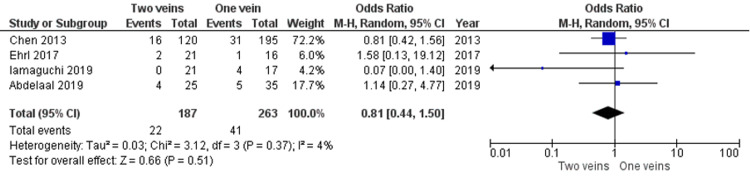
Venous congestion/thrombosis rates between one versus double venous anastomoses in ALT flaps Data reported by Chen, Ehrl, Imaguchi and Abdelaal et al. [13-15,17]. ALT: anterolateral thigh

Venous congestion/thrombosis: Four studies [13-15,17] reported venous congestion and thrombosis comparing one versus two veins, but no significant difference was seen between the two groups, as demonstrated in Figure 3 below.

**Figure 4:**
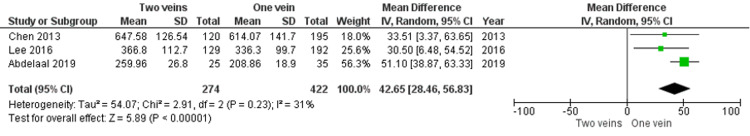
Operative time (minutes) of single versus double venous anastomoses in ALT flap reconstruction Data reported by Lee, Abdelaal and Chenet al. [12,15,17]. ALT: anterolateral thigh

Operative time: The operative time was significantly lower in the single venous anastomosis group, as reported in Figure 4, for ALT free-flap reconstruction [12,15,17]. A statistically significant mean difference (p<0.05) was evidenced favouring the use of one vein.

**Figure 5:**
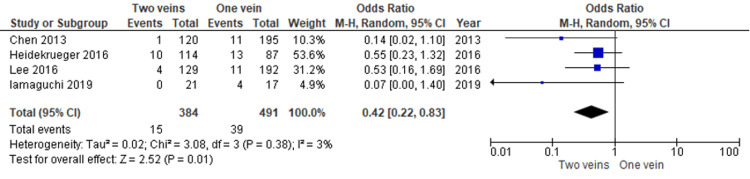
Flap take-back due to venous congestion; significantly lower rate in the double venous anastomosis group (p = 0.01) Data reported by Chen, Heidekrueger, Lee and Iamaguchi et al. [12,14,16,17].

Flap take back: The flap take-back rate was significantly lower in the double venous anastomosis group [12,14,16,17] as demonstrated in Figure 5 with a significant P value (P = 0.01).

Secondary Outcome Measures

Miscellaneous complications: Additional complications reported included partial dehiscence and partial flap loss, which have been referenced as 11.4% versus 12% in one and two veins, respectively, by Abdelaal [15]. Chen [17] reports a partial flap loss rate of 7.2% (one vein) and 4.2% (two veins). Ehrl [13] and Heidekrueger [16] did not have any incidences of partial flap loss in either group. The rates of haematoma were comparable in the study by Chen [17], 5.1% (one vein) and 5% (two veins) for ALT flaps. Ehrl [13] did not report any haematoma in the one vein group but had a 1% incidence in the double venous anastomosis cohort. Heidekrueger [16] had near-similar incidences of haematoma formation in each group, 6.9% (one vein) versus 5.26% (two veins), as did Lee [12], 4.2% (one vein) and 3.1% (two veins).

Methodological quality review: The Newcastle-Ottawa scale was used to assess the methodological quality of all observational studies within this review [11] (Table 2). Most studies scored poorly in the “Selection” domain due to no precise stratification method for patients undergoing single or double anastomoses. In the “Comparability” domain, however, most studies scored well, reporting both primary and secondary outcome measures homogenously to an extent. In the “Exposure” domain, studies scored poorly due to not stating the length of follow-up and no reports of whether any patients were lost to follow-up.

**Table 2:** The Newcastle-Ottawa scale assessing selection, comparability, and exposure across the eight included studies under review Newcastle-Ottawa scale [11]

Study	Selection	Comparability	Exposure
Ross et al [8]	***	**	*
Lee et al [12]	***	**	*
Ehrl et al [13]	***	**	**
Iamaguchi et al [14]	**	**	*
Abdelaal et al [15]	***	**	**
Heidekrueger et al [16]	***	**	**
Chen et al [17]	**	**	**
Lin et al [18]	**	**	*

Discussion

There are many debatable factors regarding whether one or two venous anastomoses are preferred in ALT flap reconstruction. Surgeons who advocate the use of a single vein stress the importance of lower operative times, better use of resources and reduced risk of thrombosis from decreased blood flow velocity [5-7], whereas surgeons who encourage the use of two veins believe this reduces the risk of venous failure and mitigates flap compromise [8,9].

This review demonstrated a significantly lower flap take-back rate for a double venous anastomosis and a shorter operative time. Other reported measures, including venous congestion and flap loss, showed an insignificant difference (P>0.05).

Abdelaal [15] and Lin et al. [18] advocate using a single vein anastomosis, as there were no significant differences in most of their outcome measures when comparing one versus two veins. Outcome measures included flap loss and complications such as infection and venous thrombosis, none of which demonstrated any statistical differences.

In contrast, Ross [8], Lee [12], Iamaguchi [14], Chen [17] and Sun et al. [19] demonstrated double venous anastomosis as a superior option. When reviewing venous congestion, Iamaguchi et al. [14] found a statistical difference favouring the double vein cohort. Lee et al. [12] found a significant difference in operative time favouring single vein anastomosis, whereas the length of hospital stay differed in preference of the double vein group, which had an average shorter inpatient duration. When looking at patients taken back to theatre, Chen et al. [17] found that, in general, there was no significant difference between the groups; however, when vascular insufficiency was analysed, there was a considerable difference between the groups, with a higher proportion of these being in the single vein group. 

Ehrl [13] and Heidekrueger et al. [16] reported, although the success of the flap was independent of the number of anastomoses used, they still suggested performing a double vein anastomosis. They both demonstrated no statistical difference when measuring surgical complications, specifically venous thrombosis rates.

Riot et al. [5] reviewed all free flaps in general; they found that using two veins decreased the risk of flap failure, venous thrombosis and the need for revision surgery. This meta-analysis has deduced comparable results for lower flap take-back rates in the double vein group. The authors of this review found no significant difference between venous thrombosis rates and the risk of flap loss. In addition, Chaput et al. [20] favoured double vein anastomosis, as there was a decrease in venous thrombosis and the need for surgical revision. Chaput et al. [20] identified that performing a two-vein anastomosis increased the operative time by 30 minutes. Analysing the results of this study, although the operative time is significantly less when performing a single venous anastomosis, the authors would still recommend double venous anastomosis be performed. Doing so will decrease the rate of return to theatre, which will inherently reduce the overall cost.

## Conclusions

The ALT flap continues to be an effective reconstructive option; however, the microvascular dilemma of one versus two veins continues to divide opinions. Primary outcome measures showed comparable results between one versus two venous anastomoses for flap loss rate, venous congestion, and thrombosis, however, the flap take-back rate was lower in the double vein group. Secondary outcome measures, including partial flap loss and haematoma formation, showed no significant difference between the groups. This review suggests that the flap take-back rate is significantly less when anastomosing two veins, and the authors recommend that utilising a second vein can circumvent the caveat of venous compromise. More high-quality studies are needed to further the current evidence base.

## Appendices

**Table 3:** PRISMA checklist: addressing the introduction, methods, results and discussion sections of this report

Section and Topic	Item #	Checklist item	Location where item is reported
TITLE			
Title	1	Identify the report as a systematic review.	Page 1
ABSTRACT			
Abstract	2	See the PRISMA 2020 for Abstracts checklist.	Page 9
INTRODUCTION			
Rationale	3	Describe the rationale for the review in the context of existing knowledge.	Page 1
Objectives	4	Provide an explicit statement of the objective(s) or question(s) the review addresses.	Page 1
METHODS			
Eligibility criteria	5	Specify the inclusion and exclusion criteria for the review and how studies were grouped for the syntheses.	Page 1
Information sources	6	Specify all databases, registers, websites, organisations, reference lists and other sources searched or consulted to identify studies. Specify the date when each source was last searched or consulted.	Page 2
Search strategy	7	Present the full search strategies for all databases, registers and websites, including any filters and limits used.	Page 2
Selection process	8	Specify the methods used to decide whether a study met the inclusion criteria of the review, including how many reviewers screened each record and each report retrieved, whether they worked independently, and if applicable, details of automation tools used in the process.	Page 2
Data collection process	9	Specify the methods used to collect data from reports, including how many reviewers collected data from each report, whether they worked independently, any processes for obtaining or confirming data from study investigators, and if applicable, details of automation tools used in the process.	Page 2
Data items	10a	List and define all outcomes for which data were sought. Specify whether all results that were compatible with each outcome domain in each study were sought (e.g. for all measures, time points, analyses), and if not, the methods used to decide which results to collect.	Page 2
	10b	List and define all other variables for which data were sought (e.g. participant and intervention characteristics, funding sources). Describe any assumptions made about any missing or unclear information.	Page 2
Study risk of bias assessment	11	Specify the methods used to assess risk of bias in the included studies, including details of the tool(s) used, how many reviewers assessed each study and whether they worked independently, and if applicable, details of automation tools used in the process.	Page 2
Effect measures	12	Specify for each outcome the effect measure(s) (e.g. risk ratio, mean difference) used in the synthesis or presentation of results.	Page 2
Synthesis methods	13a	Describe the processes used to decide which studies were eligible for each synthesis (e.g. tabulating the study intervention characteristics and comparing against the planned groups for each synthesis (item #5)).	Page 2
	13b	Describe any methods required to prepare the data for presentation or synthesis, such as handling of missing summary statistics, or data conversions.	Page 2
	13c	Describe any methods used to tabulate or visually display results of individual studies and syntheses.	Page 2
	13d	Describe any methods used to synthesize results and provide a rationale for the choice(s). If meta-analysis was performed, describe the model(s), method(s) to identify the presence and extent of statistical heterogeneity, and software package(s) used.	Page 2
	13e	Describe any methods used to explore possible causes of heterogeneity among study results (e.g. subgroup analysis, meta-regression).	Page 2
	13f	Describe any sensitivity analyses conducted to assess robustness of the synthesized results.	N/A
Reporting bias assessment	14	Describe any methods used to assess risk of bias due to missing results in a synthesis (arising from reporting biases).	Page 2
Certainty assessment	15	Describe any methods used to assess certainty (or confidence) in the body of evidence for an outcome.	Page 2
RESULTS			
Study selection	16a	Describe the results of the search and selection process, from the number of records identified in the search to the number of studies included in the review, ideally using a flow diagram.	Page 3
	16b	Cite studies that might appear to meet the inclusion criteria, but which were excluded, and explain why they were excluded.	Page 4
Study characteristics	17	Cite each included study and present its characteristics.	Page 4
Risk of bias in studies	18	Present assessments of risk of bias for each included study.	Page 6
Results of individual studies	19	For all outcomes, present, for each study: (a) summary statistics for each group (where appropriate) and (b) an effect estimate and its precision (e.g. confidence/credible interval), ideally using structured tables or plots.	Page 4 & 5
Results of syntheses	20a	For each synthesis, briefly summarise the characteristics and risk of bias among contributing studies.	Page 4 & 5
	20b	Present results of all statistical syntheses conducted. If meta-analysis was done, present for each the summary estimate and its precision (e.g. confidence/credible interval) and measures of statistical heterogeneity. If comparing groups, describe the direction of the effect.	Page 4 & 5
	20c	Present results of all investigations of possible causes of heterogeneity among study results.	Page 4 & 5
	20d	Present results of all sensitivity analyses conducted to assess the robustness of the synthesized results.	Page 4 & 5
Reporting biases	21	Present assessments of risk of bias due to missing results (arising from reporting biases) for each synthesis assessed.	Page 4 & 5
Certainty of evidence	22	Present assessments of certainty (or confidence) in the body of evidence for each outcome assessed.	Page 4 & 5
DISCUSSION			
Discussion	23a	Provide a general interpretation of the results in the context of other evidence.	Page 6
	23b	Discuss any limitations of the evidence included in the review.	Page 6
	23c	Discuss any limitations of the review processes used.	Page 6
	23d	Discuss implications of the results for practice, policy, and future research.	Page 6
OTHER INFORMATION			
Registration and protocol	24a	Provide registration information for the review, including register name and registration number, or state that the review was not registered.	Page 1
	24b	Indicate where the review protocol can be accessed, or state that a protocol was not prepared.	Page 1
	24c	Describe and explain any amendments to information provided at registration or in the protocol.	Page 1
Support	25	Describe sources of financial or non-financial support for the review, and the role of the funders or sponsors in the review.	Page 9
Competing interests	26	Declare any competing interests of review authors.	Page 9
Availability of data, code and other materials	27	Report which of the following are publicly available and where they can be found: template data collection forms; data extracted from included studies; data used for all analyses; analytic code; any other materials used in the review.	Page 4 & 5

**Table 4:** PRISMA abstract checklist

Section and Topic	Item #	Checklist item	Reported (Yes/No)
TITLE			
Title	1	Identify the report as a systematic review.	Yes
BACKGROUND			
Objectives	2	Provide an explicit statement of the main objective(s) or question(s) the review addresses.	Yes
METHODS			
Eligibility criteria	3	Specify the inclusion and exclusion criteria for the review.	Yes
Information sources	4	Specify the information sources (e.g. databases, registers) used to identify studies and the date when each was last searched.	Yes
Risk of bias	5	Specify the methods used to assess risk of bias in the included studies.	Yes
Synthesis of results	6	Specify the methods used to present and synthesise results.	Yes
RESULTS			
Included studies	7	Give the total number of included studies and participants and summarise relevant characteristics of studies.	Yes
Synthesis of results	8	Present results for main outcomes, preferably indicating the number of included studies and participants for each. If meta-analysis was done, report the summary estimate and confidence/credible interval. If comparing groups, indicate the direction of the effect (i.e. which group is favoured).	Yes
DISCUSSION			
Limitations of evidence	9	Provide a brief summary of the limitations of the evidence included in the review (e.g. study risk of bias, inconsistency and imprecision).	Yes
Interpretation	10	Provide a general interpretation of the results and important implications.	Yes
OTHER			
Funding	11	Specify the primary source of funding for the review.	N/A
Registration	12	Provide the register name and registration number.	N/A
